# Impact of body mass index on left atrial dimension in HOCM patients

**DOI:** 10.1515/med-2021-0224

**Published:** 2021-01-27

**Authors:** Yue Zhou, Miao Yu, Jingang Cui, Shengwen Liu, Jiansong Yuan, Shubin Qiao

**Affiliations:** Department of Cardiology, State Key Laboratory of Cardiovascular Disease, Fuwai Hospital, National Center for Cardiovascular Diseases, Chinese Academy of Medical Sciences and Peking Union Medical College, No. 167 Beilishi Road, Xicheng District, Beijing 100037, China; Department of Cardiology, The Second Affiliated Hospital of Nanchang University, Nanchang, China

**Keywords:** body mass index, obesity, left atrial diameter, atrial fibrillation, hypertrophic obstructive cardiomyopathy

## Abstract

**Background:**

Substantial studies have demonstrated that left atrial (LA) enlargement was a robust predictor of atrial fibrillation (AF) and obesity was a modifiable risk factor for cardiovascular diseases. However, the role of body mass index (BMI) on LA dimension in hypertrophic obstructive cardiomyopathy (HOCM) remains unclear.

**Methods:**

A total of 423 HOCM patients (average BMI 25.4 ± 3.4 kg/m^2^) were recruited for our study. Participants were stratified into three groups based on BMI: normal weight (BMI < 23 kg/m^2^), overweight (BMI 23–27.5 kg/m^2^), and obesity (BMI ≥ 27.5 kg/m^2^).

**Results:**

Compared with normal weight, patients with obesity had significantly lower prevalence of syncope (*p* = 0.007) and moderate or severe mitral regurgitation (*p* = 0.014), and serum NT-proBNP (*p* = 0.004). Multiple linear regression analysis indicated that BMI (*β* = 0.328, *p* < 0.001), log NT-proBNP (*β* = 0.308, *p* < 0.001), presence of AF (*β* = 0.209, *p* = 0.001), and left ventricular diastolic diameter index (*β* = 0.142, *p* = 0.019) were independently related with LA diameter. However, BMI was not an independent predictor of the presence of AF on multivariable binary logistical regression analysis.

**Conclusions:**

BMI was independently associated with LA diameter; however, it was not an independent predictor of prevalence of AF. These results suggest that BMI may promote incidence of AF through LA enlargement in HOCM.

## 1 Introduction

Hypertrophic cardiomyopathy (HCM), an inherited heart disease affecting about 2‰ of the general population, is the most common cause of sudden cardiac death (SCD) in individuals less than 35 years old. It is mainly an autosomal dominant disease and caused by more than 1,400 mutations in 11 or more genes encoding cardiac sarcomere proteins [1]. HCM is characterized by unexplained asymmetric left ventricular hypertrophy (LVH), myocyte and myofibrillar disarray, interstitial fibrosis, and medial hypertrophy of small coronary arteries [2,3]. Approximately, two-thirds of patients with HCM are presented with obstruction of the left ventricular outflow tract (LVOT) at rest or provocation [4]. The large genetic and phenotypic heterogeneity of HCM lead to diverse clinical manifestations, ranging from asymptomatic status with normal life expectancy to progressive heart failure, atrial fibrillation (AF), and sudden death [5,6].

With an estimated prevalence of 20%, atrial fibrillation is the most common arrhythmia in patients with HCM, potentially increasing the risk of embolic stroke and aggravating progressive heart failure [1,7,8,9]. Therefore, early recognition of predisposing factors to AF has important implications for longitudinal surveillance and timely prophylactic interventions and management strategies in patients with HCM. Several studies have suggested that left atrial enlargement is a robust predictor of AF and cardiovascular events in patients with HCM and general population [10,11,12,13,14,15,16].

Obesity, always assessed by body mass index (BMI), is an increasing worldwide public health problem and is a modifiable risk factor of various cardiovascular diseases, such as hypertension, coronary artery disease (CAD), heart failure, and so on [17,18]. Prior studies have also demonstrated that obesity is independently correlated with larger left ventricular mass (LVM), lower exercise tolerance, and worse hemodynamics in patients with HCM [19,20,21]. In addition, obesity and elevated BMI have been shown to independently increase risk of AF [22,23,24]. Furthermore, The Framingham Heart Study and MONICA/KORA Study indicated that excess AF risk associated with obesity may be mediated by left atrial enlargement [25,26]. However, there are scanty studies performed to evaluate the relationship between BMI and left atrial dimension in patients with hypertrophic obstructive cardiomyopathy (HOCM). Hence, we sought to clarify the association between BMI and left atrial size with a large cohort of HOCM patients. Because of its tomographic high spatial resolution, cardiovascular magnetic resonance (CMR) imaging was applied for the diagnosis and structural characteristics at the clinical course of HCM [27].

## 2 Material and methods

### 2.1 Study population

The protocol of our study was approved by the Ethics Committee of Fuwai Hospital (Beijing, China) and complied with the Declaration of Helsinki. The informed consents were written by all participants.

**Figure 1:**
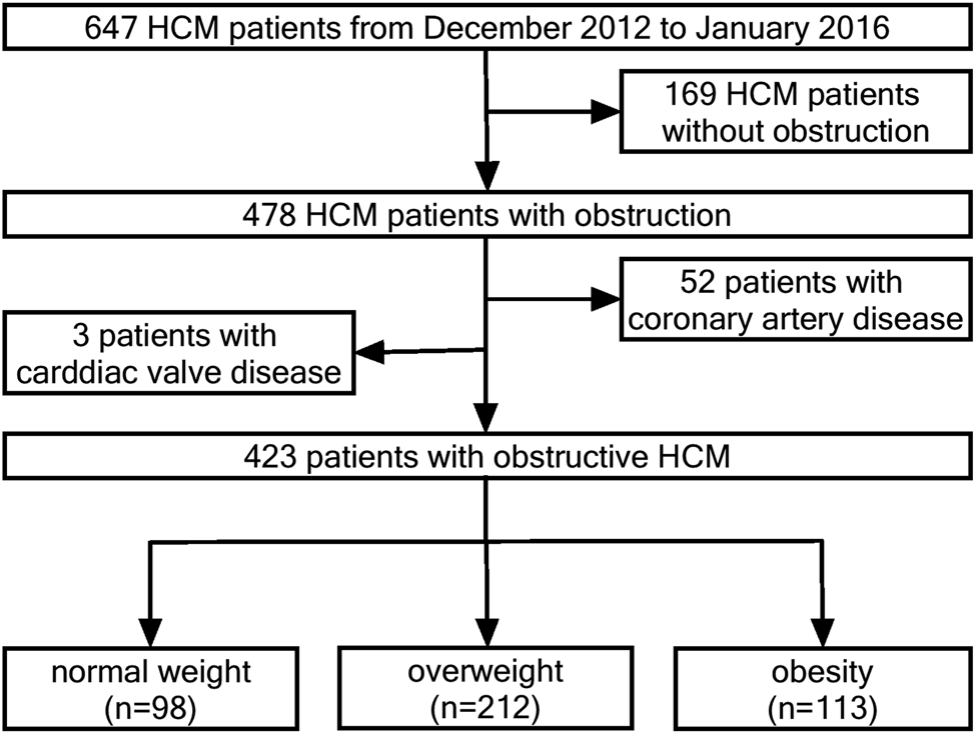
Flowchart of patient inclusion in this study. HCM, hypertrophic cardiomyopathy.

Consecutive patients with HOCM who were evaluated at Fuwai Hospital from December 2012 to January 2016 were recruited for our study. The diagnosis of HOCM was complied with the recommendation of European Society of Cardiology (ESC) [7]. Demographics, comorbidities, medical history, physical examination, blood tests, 12-lead electrocardiography, 24-h ambulatory Hotler monitoring, and echocardiographic and CMR characteristics were collected at the course of evaluation. Patients with valvular heart disease, CAD (coronary artery stenosis >50% on coronary angiography or coronary computer tomography angiography, old myocardial infarction, percutaneous coronary intervention, or coronary artery bypass surgery), concomitant neoplasma, infection, renal dysfunction (defined as estimated glomerular filtration rate <60 mL/min/1.73 m^2^), connective tissue disease, or pregnancy were excluded. In addition, subjects with a history of alcohol septal ablation, septal myectomy, or permanent mechanical device implantation were also excluded. Finally, 423 patients with HOCM were included in the present study (Figure 1). BMI was calculated as weight/(height·height) and presented with kg/m^2^. All participants were stratified into three groups based on BMI: normal weight (BMI < 23 kg/m^2^), overweight (BMI 23–27.5 kg/m^2^), and obesity (BMI ≥ 27.5 kg/m^2^), according to Asian BMI classification [28].

### 2.2 Echocardiography

According to the clinical recommendations of the American Society of Echocardiography, transthoracic echocardiography (TTE) was performed using an iE33 Color Doppler Ultrasound System (Philips Healthcare, Andover, MA, USA). The resting LVOT pressure gradient (LVOTPG) of all patients were detected by continuous-wave echocardiography, while provoking LVOTPG was only determined in those patients with a LVOTPG < 50 mm Hg at rest. Color Doppler flow imaging was conducted to assess the severity of mitral regurgitation (MR) semiquantitatively, ranked from mild to severe, in accordance with the guidelines of American Society of Echocardiography [29].

### 2.3 CMR imaging

CMR imaging was performed on a 1.5-T speed clinical scanner (Magnetom Avanto; Siemens Medical Solutions, Erlangen, Germany), with echocardiographic gating and breath holding. The imaging protocol and analysis have been described previously [30]. Left atrial anteroposterior diameter (LAD), left ventricular end-diastole volume (LVEDV), left ventricular end-systole volume (LVESV), stroke volume (SV), cardiac output (CO), left ventricular ejection fraction (LVEF), and LVM were then calculated in standard manner. All those measurements were indexed to body surface area (BSA), except for LVEF. The left ventricular end-diastole diameter (LVEDD) and maximal wall thickness (MWT) were determined in the short-axis view at end-diastole.

### 2.4 Blood measurements

Fasting venous blood samples of all recruited subjects were collected within 2 days of TTE and 1 week of CMR examination. Then all samples were analyzed by medical technologist unknown of any clinical information of the studied patients in clinical laboratory of Fuwai hospital immediately. The estimated glomerular filtration rate (eGFR, mL/min/1.73 m^2^) was derived from the Chronic Kidney Disease Epidemiology Collaboration (CKD-EPI) equation. Dyslipidemia was defined as those with serum LDL-C ≥ 3.37 mmol/L, TG ≥ 1.70 mmol/L, or use of lipid-lowering drugs.

### 2.5 Statistical analysis

The values were expressed as mean ± standard deviation (SD) or median (interquartile range [IQR]) for continuous variables, according to their distribution. Categorical variables were presented as frequencies (percentages). Comparisons of continuous variables were assessed with independent student’s *t* test, Mann–Whitney *U* test, one-way analysis of variance (followed by the Dunnett’s *t* test for multiple groups) or Kruskal–Wallis *H* test appropriately. Differences in categorical variables were evaluated with *χ* ^2^ test or Fisher’s exact test (as appropriate). Pearson’s correlation test or Spearman’s correlation test was used to examine the simple correlation between two continuous variables properly. In order to obtain normal distribution, logarithmic transformation was applied for the analysis of N-terminal pro-B-type natriuretic peptide (NT-proBNP). To estimate the relationship between BMI and LA dimension in HOCM patients, stepwise multiple linear regression analysis (*p* value threshold to enter ≤0.05; to remove, ≤0.10) was conducted by adjusting for potential confounding factors influencing LA dimension. Multivariable binary logistic regression analysis using backward elimination was performed to identify independent predictors of the presence of AF. The predicting variables included those with a *p* < 0.1 in the univariable analysis or those published on previous literature. A two-tailed *p* value <0.05 was considered as statistical significance. All analyses of data were applied with SPSS version 25.0 software (SPSS Inc, Chicago, IL, USA).

## 3 Results

A total of 423 patients with HOCM were included in the present study (mean age 48.2 ± 12.2 years, range 18–74 years), comprising 249 males (58.9%) and 174 females (41.1%). Average BMI was 25.4 ± 3.4 kg/m^2^, ranging from 13.0 to 36.4 kg/m^2^. Ninety-eight patients (23.2%, male 42.9%) were in normal weight range (BMI < 23 kg/m^2^, mean 21.0 ± 1.7 kg/m^2^), 212 (50.1%, male 61.8%) were overweight (BMI 23–27.5 kg/m^2^, mean 25.3 ± 1.3 kg/m^2^), and 113 (26.7%, male 67.3%) were obesity (BMI ≥ 27.5 kg/m^2^, mean 29.6 ± 1.9 kg/m^2^). Overall, 355 patients (83.9%) were detected that resting LVOTPG was more than 30 mm Hg. The prevalence of AF in these patients was 13.5%.

**Figure 2:**
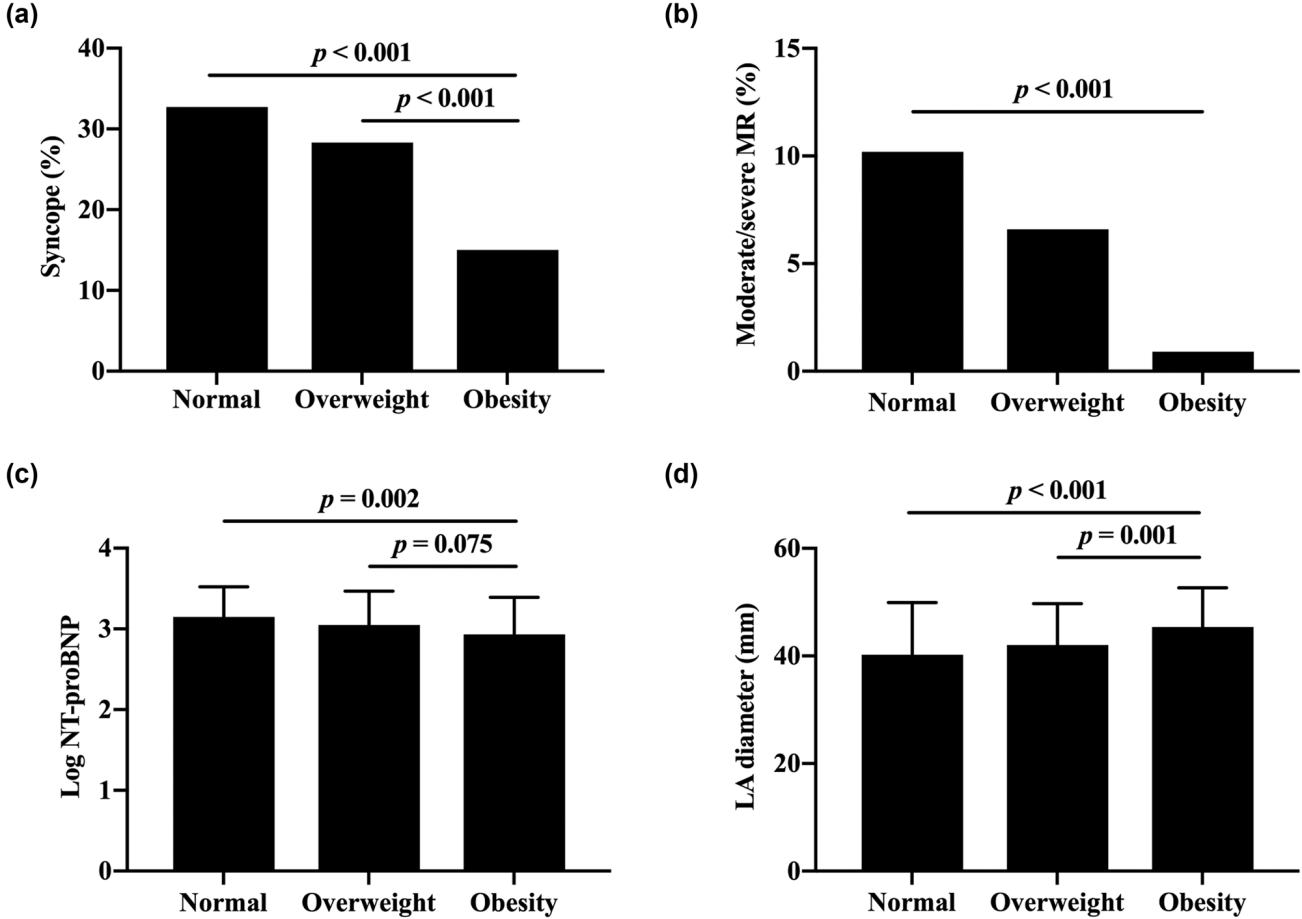
Prevalence of syncope (a) and moderate or severe MR (b), log NT-proBNP (c), and LA diameter (d) in each subgroup. MR, mitral regurgitation; NT-proBNP, N-terminal pro-B-type natriuretic peptide; LA, left atrial.

The demographic and clinical characteristics of studied population stratified by BMI are summarized in Table 1. The prevalence of traditional cardiovascular risk factors (*p* < 0.001), serum uric acid (SUA, *p* = 0.001), and high-sensitivity C-reactive protein (hs-CRP, *p* < 0.001) increased disproportionally among subgroups. Compared with patients with overweight, there was an insignificantly rising tendency of AF in normal weight and obesity (9.9, 14.3, and 19.5%, respectively; *p* = 0.054). However, incidence of syncope (*p* = 0.007; Figure 2a) and moderate or severe MR (*p* = 0.014; Figure 2b), and log NT-proBNP (*p* = 0.004; Figure 2c) were significantly lower in obese patients. Moreover, obese patients had significantly larger LAD compared with normal weight and overweight (40.2 ± 9.7, 42.0 ± 7.7, 45.4 ± 7.3, respectively; *p* < 0.001; Figure 2d). With respect to eGFR, New York Heart Association (NYHA) ≥ III, family history of HCM and SCD, nonsustained ventricular tachycardia (NSVT), MWT ≥ 30 mm, and resting LVOTPG ≥ 30 mm Hg, LVESV index, LVEDV index, SV index, CO index, and LVM index, there were no significant differences among different subgroups.

**Table 1:** Demographic and clinical characteristics of patients with hypertrophic obstructive cardiomyopathy stratified by BMI

Variable	Overall	Normal	Overweight	Obesity	*p* value
		BMI < 23 kg/m^2^	BMI 23–27.5 kg/m^2^	BMI ≥ 27.5 kg/m^2^	
*n*	423	98	212	113	
Male, *n* (%)	249 (58.9%)	42 (42.9%)	131 (61.8%)	76 (67.3%)	0.001
Age (years)	48.2 ± 12.2	45.7 ± 14.5	48.6 ± 11.8	49.6 ± 10.5	0.052
BMI (kg/m^2^)	25.4 ± 3.4	21.0 ± 1.7	25.3 ± 1.3	29.6 ± 1.9	<0.001
BSA (m^2^)	1.76 ± 0.20	1.57 ± 0.14	1.76 ± 0.16	1.92 ± 0.15	<0.001
Systolic blood pressure (mm Hg)	118.7 ± 16.8	113.1 ± 16.3	119.1 ± 16.8	122.8 ± 16.2	<0.001
Diastolic blood pressure (mm Hg)	72.5 ± 10.3	68.0 ± 10.0	73.4 ± 10.1	74.6 ± 9.9	<0.001
Heart rate (beats/min)	71.8 ± 10.8	72.7 ± 11.2	71.4 ± 10.2	71.7 ± 11.5	0.616
NYHA functional class III or IV, *n* (%)	145 (34.3%)	34 (34.7%)	72 (34%)	39 (34.5%)	0.990
Chest pain, *n* (%)	195 (46.1%)	46 (46.9%)	91 (42.9%)	58 (51.3%)	0.345
Palpitation, *n* (%)	138 (32.6%)	32 (32.7%)	65 (30.7%)	41 (36.3%)	0.589
Family history of HCM, *n* (%)	56 (13.2%)	14 (14.3%)	29 (13.7%)	13 (11.5%)	0.808
Atrial fibrillation, *n* (%)	57 (13.5%)	14 (14.3%)	21 (9.9%)	22 (19.5%)	0.054
**Risk factor for SCD**					
Family history of SCD, *n* (%)	22 (5.2%)	7 (7.1%)	10 (4.7%)	5 (4.4%)	0.610
Syncope, *n* (%)	109 (25.8%)	32 (32.7%)	60 (28.3%)	17 (15.0%)	0.007
Maximum wall thickness ≥ 30 mm, *n* (%)	74 (17.5%)	15 (15.3%)	41 (19.3%)	18 ( 15.9%)	0.601
Resting LVOTPG ≥ 30 mm Hg, *n* (%)	355 (83.9%)	86 (87.8%)	177 (83.5%)	92 (81.4%)	0.444
Nonsustained VT^a^, *n* (%)	42 (19.7%)	11 (23.4%)	22 (19.1%)	9 (17.6%)	0.753
**Traditional cardiovascular risk factor**					
Hypertension, *n* (%)	140 (33.1%)	7 (7.1%)	76 (35.8%)	57 (50.4%)	<0.001
Diabetes mellitus, *n* (%)	27 (6.4%)	0 (0%)	11 (5.2%)	16 (14.2%)	<0.001
Dyslipidemia, *n* (%)	150 (35.5%)	22 (22.4%)	73 (34.4%)	55 (48.7%)	<0.001
Current smokers, *n* (%)	155 (36.6%)	19 (19.4%)	83 (39.2%)	53 (46.9%)	<0.001
**Laboratory examination**					
eGFR (mL/min/1.73 m^2^)	98.0 ± 20.0	99.8 ± 17.3	98.6 ± 22.6	95.3 ± 16.7	0.212
Serum uric acid (μmol/L)	366.7 ± 93.3	339.5 ± 81.0	368.6 ± 88.6	386.5 ± 106.2	0.001
HbA1c (%)	5.6 ± 0.7	5.5 ± 0.4	5.6 ± 0.6	5.8 ± 1.1	0.029
hs-CRP (mg/L)	1.06 (0.51–1.98)	0.65 (0.15–1.45)	1.10 (0.60–1.97)	1.25 (0.79–2.22)	<0.001
Total cholesterol (mmol/L)	4.51 ± 1.00	4.47 ± 0.90	4.51 ± 0.93	4.56 ± 1.18	0.794
Triglycerides (mmol/L)	1.59 ± 1.07	1.24 ± 0.56	1.61 ± 1.25	1.86 ± 0.96	<0.001
HDL-C (mmol/L)	1.14 ± 0.31	1.27 ± 0.34	1.13 ± 0.30	1.03 ± 0.27	<0.001
LDL-C (mmol/L)	2.85 ± 0.86	2.77 ± 0.73	2.85 ± 0.84	2.90 ± 0.99	0.502
cTNI (ng/mL)	0.022 (0.009–0.042)	0.019 (0.010–0.048)	0.022 (0.007–0.042)	0.022 (0.013–0.041)	0.907
NT-proBNP (pmol/L)	1183.0 (635.5–2270.6)	1488.0 (811.4–2597.5)	1292.3 (734.7–2344.6)	968.9 (415.4–1717.7)	0.004
Log NT-proBNP	3.04 ± 0.42	3.15 ± 0.37	3.05 ± 0.42	2.93 ± 0.46	0.002
**Medications**					
Beta-blockers, *n* (%)	318 (75.2)	75 (76.5)	159 (75.0)	84 (74.3)	0.931
Nondihydropyridine CCB, *n* (%)	68 (16.1)	11 (11.2)	37 (17.5)	20 (17.7)	0.328
Amiodarone, *n* (%)	11 (2.6)	5 (5.1)	3 (1.4)	3 (2.7)	0.165
ACEI/ARB, *n* (%)	48 (11.3)	5 (5.1)	23 (10.8)	20 (17.7)	0.015
Statins, *n* (%)	50 (11.8)	8 (8.2)	29 (13.7)	13 (11.5)	0.373
**Echocardiography**					
Systolic anterior motion, *n* (%)	384 (90.8)	90 (91.8)	195 (92.0)	99 (87.6)	0.396
Moderate or severe mitral regurgitation, *n* (%)	25 (5.9)	10 (10.2)	14 (6.6)	1 (0.9)	0.014
LVOTPG at rest (mm Hg)	70.3 ± 36.3	75.3 ± 38.8	70.6 ± 36.7	65.5 ± 32.7	0.153
LVOTPG after provocation^b^ (mm Hg)	78.6 ± 30.8	80.2 ± 43.1	81.6 ± 26.8	72.7 ± 28.5	0.430
**Cardiovascular magnetic resonance**					
Left atrial diameter (mm)	42.5 ± 8.3	40.2 ± 9.7	42.0 ± 7.7	45.4 ± 7.3	<0.001
Left atrial diameter index (mm/m^2^)	24.4 ± 5.4	25.9 ± 6.9	24.1 ± 4.9	23.8 ± 4.4	0.040
LV end-diastole diameter (mm)	46.0 ± 4.9	44.9 ± 5.3	46.2 ± 4.6	46.6 ± 4.9	0.025
Maximum wall thickness (mm)	24.5 ± 5.3	24.5 ± 5.1	24.4 ± 5.6	24.7 ± 4.9	0.915
LV ejection fraction (%)	68.4 ± 8.0	67.8 ± 8.7	68.5 ± 8.0	68.6 ± 7.6	0.722
Cardiac output (L/min)	5.9 ± 1.7	5.4 ± 1.4	5.9 ± 1.8	6.4 ± 1.7	<0.001
LV end-systole volume index (mL/m^2^)	23.5 ± 9.8	24.9 ± 11.3	23.1 ± 9.0	23.2 ± 10.0	0.290
LV end-diastole volume index (mL/m^2^)	72.2 ± 16.6	73.9 ± 17.2	72.2 ± 16.9	70.8 ± 15.3	0.409
Stroke volume index (mL/m^2^)	49.0 ± 11.1	49.6 ± 10.9	49.1 ± 11.8	48.4 ± 10.1	0.794
Cardiac index (L/m^2^)	3.37 ± 0.88	3.43 ± 0.87	3.37 ± 0.92	3.31 ± 0.81	0.630
LV mass index (g/m^2^)	89.9 ± 36.2	89.7 ± 38.0	88.8 ± 35.6	92.4 ± 36.1	0.696

Data are expressed as mean ± SD, number (percentage), or median (interquartile range).

BMI: body mass index, BSA: body surface area, NYHA: New York Heart Association, HCM: hypertrophic cardiomyopathy, SCD: sudden cardiac death, LVOTPG: left ventricular outflow tract pressure gradient, VT: ventricular tachycardia, eGFR: estimated glomerular filtration rate, HbA1c: glycated hemoglobin, hs-CRP: high-sensitivity C-reactive protein, HDL-C: high-density lipoprotein cholesterol, LDL-C: low-density lipoprotein cholesterol, NT-proBNP: N-terminal pro-B-type natriuretic peptide, CCB: calcium channel blocker, ACEI: angiotensin converting enzyme inhibitor, ARB: angiotensin receptor blocker, LV: left ventricular.

a Ambulatory 24-h Holter monitoring data were available in 213 of the 423 participants.

b Provoking LVOTPG data were obtained in 103 patients.

**Figure 3:**
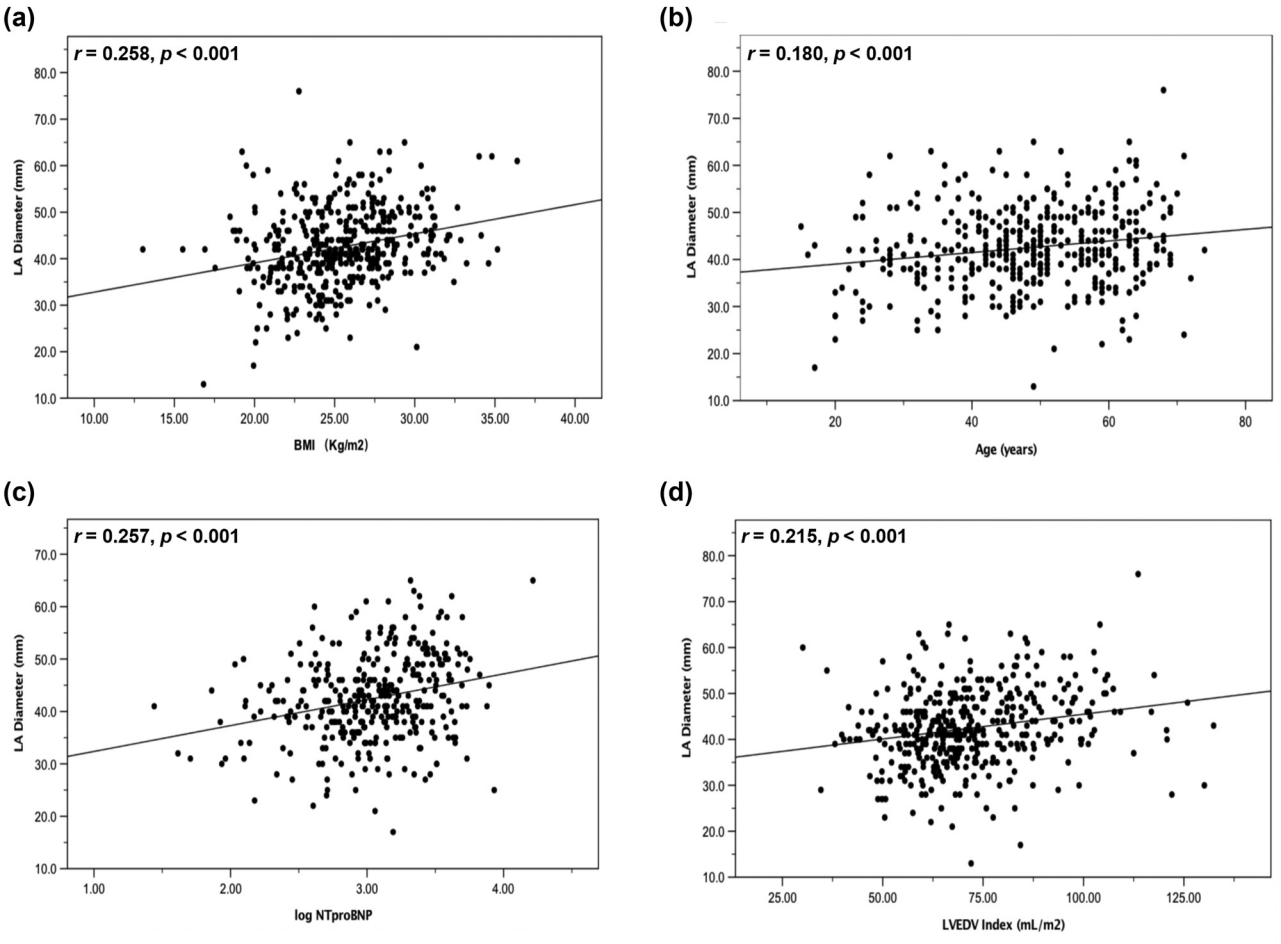
Scatter plots demonstrating the correlations between LAD and BMI (a), age (b), log NT-proBNP (c), and LVEDVI (d). LAD, left atrial diameter; BMI, body mass index; NT-proBNP, N-terminal pro-B-type natriuretic peptide; LVEDVI, left ventricular end diastolic volume index.

Table 2 shows univariable analysis of correlation between variables and BMI and LA diameter. There were significant correlations between BMI and LA diameter (*r* = 0.258, *p* < 0.001; Figure 3a). Similarly, LAD was positively correlated with age (*r* = 0.180, *p* < 0.001; Figure 3b), SUA, hs-CRP, total cholesterol (TC), low-density lipoprotein cholesterol (LDL-C), cardiac troponin I (cTNI), and log NT-proBNP (*r* = 0.257, *p* < 0.001; Figure 3c), while negatively with eGFR. In addition, significant correlations were found between LAD and provoking LVOTPG, LVEDD, LVESV index, LVEDV index (*r* = 0.215, *p* < 0.001; Figure 3d), SV index, cardiac output index, and LVM index.

**Table 2:** Univariable analysis of correlation between variables and BMI and LA diameter

Variable	Body mass index		LA diameter	
	Correlation coefficient (*r*)	*p* value	Correlation coefficient (*r*)	*p* value
Age (years)	0.123	0.012	0.180	<0.001
BMI (kg/m^2^)	—	—	0.258	<0.001
Systolic blood pressure (mm Hg)	0.203	<0.001	0.038	0.423
Diastolic blood pressure (mm Hg)	0.212	<0.001	−0.013	0.793
Heart rate (beats/min)	−0.003	0.952	0.040	0.416
eGFR (mL/min/1.73 m^2^)	−0.071	0.143	−0.115	0.018
Serum uric acid (μmol/L)	0.206	<0.001	0.170	<0.001
HbA1c (%)	0.143	0.016	−0.035	0.562
hs-CRP (mg/L)	0.202	<0.001	0.155	0.001
Total cholesterol (mmol/L)	0.040	0.410	0.140	0.004
Triglycerides (mmol/L)	0.246	<0.001	0.031	0.531
HDL-C (mmol/L)	−0.302	<0.001	−0.049	0.312
LDL-C (mmol/L)	0.067	0.166	0.146	0.003
cTNI (ng/mL)	−0.002	0.974	0.154	0.020
Log NT-proBNP	−0.189	<0.001	0.257	<0.001
LVOTG at rest (mm Hg)	−0.083	0.091	0.081	0.103
LVOTG after provocation (mm Hg)^a^	−0.017	0.867	−0.230	0.019
Left atrial diameter (mm)	0.258	<0.001	—	—
LV end-diastole diameter (mm)	0.169	<0.001	0.212	<0.001
Maximum wall thickness (mm)	0.029	0.553	0.041	0.406
LV ejection fraction (%)	0.033	0.498	−0.032	0.514
LV end-systole volume index (mL/m^2^)	−0.026	0.587	0.176	<0.001
LV end-diastole volume index (mL/m^2^)	−0.024	0.617	0.215	<0.001
Stroke volume index (mL/m^2^)	−0.001	0.988	0.212	<0.001
Cardiac index (L/m^2^)	−0.038	0.437	0.155	0.001
LV mass index (g/m^2^)	0.062	0.200	0.126	0.009

Abbreviation as in Table 1.

a Provoking LVOTG data were obtained in 103 patients.

Multiple linear regression analysis was performed to identify independent determinants of LA diameter in patients with HOCM in Table 3. BMI was independently associated with LAD (*β* = 0.328, *p* < 0.001), after adjustment for age, gender, hypertension, diabetes, dyslipidemia, usage of ACEI/ARB, eGFR, SUA, hs-CRP, TC, LDL-C, cTNI, resting LVOTPG ≥ 30 mm Hg, moderate or severe MR, systolic anterior motion (SAM), LVESV index, SV index, CO index, LVM index. Additionally, log NT-proBNP (*β* = 0.308, *p* < 0.001), presence of AF (*β* = 0.209, *p* = 0.001), and LVEDV index (*β* = 0.142, *p* = 0.019) were also independently related with LAD.

**Table 3:** Multiple linear regression analysis for the association between of LA diameter and variables in patients with hypertrophic obstructive cardiomyopathy

Variable	Standardized coefficients (β)	*p* value
Body mass index	0.328	<0.001
Log NT-proBNP	0.308	<0.001
Presence of AF	0.209	0.001
LV end-diastole volume index	0.142	0.019

Abbreviations as in Table 1, multiple *R* = 0.544, *R* ^2^ = 0.296.

Multivariable binary logistic regression analysis was conducted to reveal potential predictors of the presence of AF in patients with HOCM. As shown in Table 4, both age and LAD were independent predictors of the presence of AF in HOCM patients (OR = 1.057, *p* = 0.001; OR = 1.095, *p* < 0.001, respectively), while BMI was not. In addition, log NT-proBNP (OR = 3.205, *p* = 0.020), and LVEDD (OR = 1.092, *p* = 0.020) were also independently associated with the presence of AF, whereas resting LVOTPG predicted less likely presence of AF (OR = 0.984, *p* = 0.004).

**Table 4:** Multivariable logistic regression analysis for prediction of the presence of atrial fibrillation

Variable	OR	95% CI	*p* value
Age	1.057	1.024–1.092	0.001
Log NT-proBNP	3.205	1.197–8.586	0.020
LA diameter	1.095	1.044–1.148	<0.001
LV end-diastole diameter	1.092	1.014–1.177	0.020
Resting LVOTPG	0.984	0.973–0.995	0.004

OR: odds ratio, CI: confidence interval, NT-proBNP: N-terminal pro-B-type natriuretic peptide, LA: left atrial, LV: left ventricular, LVOTPG: left ventricular outflow tract pressure gradient.

## 4 Discussion

AF is a common sequela for patients with HCM and associated with worse symptoms and exercise capacity, and a significantly higher risk of heart failure related mortality and embolic stroke [1,8,9,12,15]. Substantial studies have demonstrated aging and left atrial enlargement were closely related with susceptibility to AF in HCM [1,7,9,12,15,16]. Aging is an unmodifiable factor, but left atrial enlargement is not. Obesity, a traditional risk factor of cardiovascular system, has been proved to increase the risk of AF through amplifying left atrial dimension in general population [22,23,24,25,26]. However, the relationship of BMI and left atrial size in HOCM patients has not been illuminated. For the first time, our present study revealed that HOCM patients with obesity had significantly lower prevalence of syncope and moderate or severe MR, and serum NT-proBNP, compared with patients with normal weight. Furthermore, BMI, NT-proBNP, presence of AF, and LVEDVI were independently and positively associated with LA diameter. After adjusting for possible relevant variables in multivariable logistic regression analysis, age, log NT-proBNP, LAD, LVEDD, and resting LVOTPG were independent predictors of the presence of AF, whereas BMI was not.

Hypovolemia, sinus node dysfunction, complete atrioventricular block, NSVT, LVOT obstruction, and abnormal vascular reflexes are contributed to syncope in HCM [7]. The current study demonstrated that syncope and moderate or severe MR in HOCM patients with obesity was significantly lower than that in normal weight. Multiple cross-sectional studies showed that the lower BMI had greater risk of noncardiac syncope via downregulating activity of sympathetic nervous system and upregulating activity of parasympathetic nervous system in the general individuals [31]. Moreover, Jones et al. indicated that MR was independently related to lower BMI in the Strong Heart Study including 3,486 American Indian participants [32]. Hence, the predisposition of low BMI to syncope in patients with HOCM may partly be accounted for low cardiac output induced by MR and abnormal vascular reflexes induced by autonomic dysfunction. Further studies are required to confirm our assumptions. Lower level of serum B-type natriuretic peptide was associated with obesity due to adipose tissue [33]. Consistent with previous study, our study demonstrated that HOCM patients with obesity had significantly lower serum NT-proBNP.

Obesity is becoming a globally public health crisis in both children and adults, and it is an independent risk factor for cardiovascular disease and linked with dramatic comorbidities such as hypertension, type 2 diabetes mellitus, hyperuricemia, dyslipidemia, and obstructive sleep apnea [34]. Furthermore, Olivotto et al. [21] conducted a study of 275 HCM patients with a median follow-up of 3.7 years, which revealed that BMI was independently associated with the magnitude of LVM and may promote progression of heart failure symptoms. Canepa et al. [19] demonstrated that there were larger LVM, worse symptoms, lower exercise intolerance, and more labile obstructive hemodynamics in obese, compared to patients with normal weight. Additionally, a large amount of evidence has documented that obesity and elevated BMI were independently associated with AF occurrence in general individuals [22,23,24]. Wang et al. [26] showed that obesity was an important and potentially modifiable risk factor for new-onset AF. The increased risk for AF associated with obesity appeared to be mediated by LA dilation. The MONICA/KORA Study demonstrated that obesity appears to be the most important risk factor for LA enlargement during aging [25]. In agreement with these studies, our present study demonstrated that BMI was significantly and positively associated with LA size in patients with obstructive HCM on the multiple linear regression analysis model. Nevertheless, inconsistent with the previous studies, after adjustment for possible relevant variables in multivariable logistic regression analysis, BMI was no longer associated with the presence of AF, but LAD was independent predictor of AF occurrence. This difference may be potentially explained by the relatively low detection rate of AF, different study populations, and an important “intermediate” phenotype associated with BMI and AF [26]. Moreover, contrary to findings of the aforementioned literature, our results did not find the relationship between BMI and LVMI. This discrepancy may partly be contributed to a relatively low BMI of patients with HOCM, heterogeneity of patients’ features, and methodological differences. Hence, further studies including a larger number of HOCM patients with higher BMI are required to elucidate the relationship between BMI and AF occurrence and LV mass index.

The precise mechanisms underlying the relationship between BMI and LA dimension are still undetermined. Pressure and volume overload [10], inflammation, and enhanced neurohormonal activation accompanying obesity may promote LA enlargement [14,22]. Furthermore, adiposity may influence myocardial structure through enhancement of oxidative stress or lipoapoptosis [26]. In addition, autonomic dysfunction and obstructive sleep apnea in obesity individuals may also be related to cardiac structure [23,34,35]. Herein further investigations are needed to clarifying the pathophysiological mechanisms underlying the role of obesity or elevated BMI on LA dimension in patients with HOCM.

A systematic review conducted by Overvad et al. [14] manifested that patients with larger LA had a higher risk of stroke compared to those with smaller or normal LA size in sinus rhythm. LA enlargement has been considered as a strong predictor of AF [12,16], congestive heart failure, and cardiovascular mortality [10] in the general and HCM population. Hence, owing to independent and positive relationship between BMI and LA size proved in our study, weight loss or BMI management may reduce incidence of AF and common cardiovascular outcomes in HOCM. However, there is an obvious and serious divergence on the concept of LA enlargement identified with various methods and partition values. Indexing of LA dimension by body surface area (BSA) is currently recommended and most frequently used [10,25]; however, this approach may lead to an underestimation of LA remodeling, especially in obese patients [36,37]. Therefore, it is necessary to seek for an excellent method of normalization of LA volume measurements.

There are several limitations in our study. First, this was a retrospectively cross-sectional and single center study with inherent risk for different types of bias, which was difficult to confirm causal relationship. It is necessary to perform prospective cohort studies and a long-time follow-up for establishing cause and effect. Second, HOCM patients have a relatively low BMI in China. A larger number of HOCM patients with higher BMI have effectively elucidated the relationship of BMI and incidence of AF. Thirdly, LA dimension was evaluated with measurement of LA anteroposterior diameter. Although this method is simple and convenient and CMR has superior reproducibility, it is not reliably accurate because of asymmetrically shaped three-dimensional structure of LA and multiple fashion of LA enlargement [10]. Finally, the prevalence of AF was lower than that of aforementioned literature. Holter monitor should be applied repeatedly to improve the detection rate of AF.

In conclusion, BMI was independently associated with LA diameter in patients with HOCM. However, it was not an independent predictor of prevalence of AF. These results suggest that BMI may promote incidence of AF through LA enlargement. Further studies are required to verify our hypothesis.
